# Evaluation of the WHO revised criteria for classification of clinical disease severity in acute adult dengue infection

**DOI:** 10.1186/1756-0500-5-645

**Published:** 2012-11-20

**Authors:** SD Jayaratne, Vajini Atukorale, Laksiri Gomes, Thashi Chang, Tharindu Wijesinghe, Sachie Fernando, Graham S Ogg, Gathsaurie Neelika Malavige

**Affiliations:** 1 Department of Medicine, University of Sri Jayawardanapura, Nugegoda, Sri Lanka; 2 Department of Microbiology, Faculty of Medical Sciences, University of Sri Jayawardanapura, Nugeguda, Sri Lanka; 3 Department of Clinical Medicine, Faculty of Medicine, University of Colombo, Colombo, Sri Lanka; 4 MRC Human Immunology unit, Weatherall Institute of Molecular Medicine, Oxford

**Keywords:** Dengue virus infection, Shock, Warning signs, WHO guidelines, Laboratory parameters, Clinical features

## Abstract

**Background:**

The WHO guidelines were revised recently to identify patients with severe dengue (SD) early. We proceeded to determine the usefulness of the warning signs in the new WHO guidelines in predicting SD and we have also attempted to define other simple laboratory parameters that could be useful in predicting SD.

**Methods:**

Clinical and laboratory parameters were recorded in 184 patients in 2011, with confirmed dengue viral infections, admitted to a medical ward in two tertiary care hospitals in Colombo, Sri Lanka.

**Results:**

We found that the presence of 5 or more dengue warning signs were significantly (p = 0.02) associated with the development of SD (odds ratio 5.14, 95% CI = 1.312 to 20.16). The AST levels were significantly higher (p = 0.0001) in patients with abdominal pain (mean 243.5, SD ± 200.7), when compared to those who did not have abdominal pain (mean 148.5, SD ± 218.6). Lymphocyte counts <1,500 cells/mm^3^ were significantly (p = 0.005) associated with SD (odds ratio 3.367, 95% CI 1.396 to 8.123). High AST levels were also significantly associated (p < 0.0001) with SD (odds ratio 27.26, 95% CI 1.632 to 455.2). Platelet counts <20,000cells/mm^3^, were again significantly associated (p < 0.001) with severe disease (odds ratio 1.632 to 455.2, 95% CI 3.089 to 14.71). The PCR was positive in 26/84 of the patients and we found that the infecting serotype was DEN-1 in all 26 patients.

**Conclusions:**

The presence of 5 or more warning signs appears to be a predictor of SD. Lymphocyte counts <1,500 cells/mm^3^, platelet counts <20,000/mm^3^ and raised AST levels were associated with SD and could be used to help identify patients who are likely to develop SD.

## Background

Dengue viral infections have become one of the most important mosquito borne viral infections in the world and is one of the major emerging infectious diseases. It is estimated that 2.1 million cases of dengue haemorrhagic fever (DHF)/dengue shock syndrome (DSS) occur every year resulting in 21,000 deaths [1]. Infection with the dengue virus (DV), may manifest as asymptomatic or a mild febrile illness, dengue fever (DF), dengue haemorrhagic fever (DHF) or dengue shock syndrome (DSS) which can be fatal [2,3].

Sri Lanka has been affected by epidemics of DHF for over 2 decades. Dengue viral infections have been endemic in Sri Lanka since the mid 1960s, which was when the first cases of DF/DHF were reported [4,5]. Although the Sri Lankan population had been exposed to the virus for decades, severe forms of dengue infection (DHF/DSS) were rare before 1989. The first large outbreak of DHF occurred in 1989 with 206 clinically diagnosed cases of dengue and 20 deaths (case fatality rate 9.8%) and in 1990, 1080 cases with 60 deaths. Since then the number of cases of dengue have been rising each year and in recent years dengue ranks highest in mortality in mosquito borne infection in Sri Lanka [6]. Sri Lanka experienced the worst ever dengue outbreak in years 2009 and 2010 with 35000 cases in year 2009 with 246 deaths and 34105 cases and 229 deaths in the year 2010 [7]. Not only is the incidence of dengue infections rising in Sri Lanka, but also the infection is spreading to all parts of Sri Lanka.

Case fatalities due to dengue infection are around 1-5% depending on the country and epidemic [8]. Early detection of shock and other complications and supportive therapy has been shown to reduce the morbidity and mortality [8-10]. There was much criticism regarding the WHO 1997 dengue classification guidelines, in particular that it underestimated many patients who developed shock and severe dengue [11,12]. In addition, many pointed out the need to standardize the dengue diagnostic criteria in order to implement better patient management guidelines [13]. Therefore, following the observations of a large multi-centre study, which was conducted in South East Asia and Latin America, the WHO revised their guidelines in 2009 [13]. Accordingly the WHO changed their clinical classification of acute dengue as probable dengue, dengue with warning signs and severe dengue, which were thought to be more simple and easier to understand [8,14]. Furthermore, the revised guidelines have been shown to be more sensitive and specific in detecting patients with severe dengue than the traditional guidelines [15]. The warning signs were devised in order to help health care professionals to decide who needs hospital admission and more intensive monitoring during epidemics of dengue infection, without the need for extensive laboratory investigations [14].

During dengue epidemics in Sri Lanka, very large numbers of patients are admitted every day and due to the scarcity of resources, close monitoring of all dengue patients becomes impossible. Therefore, especially once patients are admitted to hospital, triage of patients based on the presence of certain warning signs would be crucial to decide the patients who are more likely to develop severe disease. Such warning signs should be able to help the medical personnel to make decisions regarding those who need more intensive care. Therefore, we proceeded to determine the usefulness of the warning signs in the new WHO guidelines in predicting severe dengue and we have also attempted to define other simple laboratory parameters that could be useful in predicting severe dengue. In this study, we have characterized the clinical and laboratory features in a large cohort of adult dengue patients with acute infection.

## Methods

### Patients

184 adult patients with clinical features suggestive of dengue infection who were admitted to a general medical ward in two tertiary care hospitals in Colombo during the year 2011, were enrolled in the study following informed written consent. The study was approved by the Ethical Review Committee of the University of Sri Jayawardanapura. Clinical features such the presence of dengue warning signs, blood pressure, pulse rate and volume, general status of the patient, presence of any bleeding manifestations and presence of any possible fluid accumulation in the pleural cavity and abdomen were monitored several times a day from the time of admission to hospital, until they were discharged. Laboratory investigations such as the full blood count, AST, ALT were done several times a day in all patients until they were discharged from hospital. Serum electrolytes, coagulation profiles, chest radiography and ultrasound scans of the abdomen were only done in selected patients who developed severe dengue due to resource limitations. All patients included in our study had an increase in their haematocrits (HCTs), which was defined as a HCT increase of ≥20% from the baseline of patient or population of the same age and thrombocytopenia (100,000 cells/mm^3^ or less). These patients were classified as having dengue with warning signs (moderately severe dengue) and severe dengue according to the 2009 WHO guidelines [8]. Based on the 2009 WHO diagnostic criteria, shock was defined as lowering of pulse pressure to 20 mmHg or less or the presence of signs of poor capillary perfusion (cold extremities, poor capillary refill or a rapid pulse rate) [8]. Accordingly, 38 (20.6%) patients were classified as having shock. 2 other patients who did not have shock were classified as having severe disease as their liver enzymes, alanine transaminase (ALT) levels and aspartate transaminase (AST) levels, were >1000 IU. Accordingly, 40 patients were classified as having severe dengue and 144 patients were classified has having dengue with warning signs (non severe dengue).

### Serology

Acute dengue infection was confirmed by testing the serum samples which were collected after day 6 of illness with a commercial capture-IgM and IgG enzyme-linked immunosorbent assay (ELISA) (Panbio, Brisbane, Australia). The ELISA was performed and the results were interpreted according to the manufacturer’s instructions. This ELISA assay has been validated as both sensitive and specific for primary and secondary dengue virus infections [16,17]. The combined use of both IgM and IgG been shown to have a sensitivity of 99% and a specificity of 96% to distinguish between primary and secondary dengue infections [17]. Patients who only had dengue virus specific IgM were classified as having a primary dengue (PD) infection while those who had a positive result for both IgM and IgG were classified as having a secondary dengue (SD infection) [8]. Accordingly, 46 (25%) of the individuals were classified has having a PD infection. However, the classification of patients to be having primary dengue or secondary dengue based on this test alone may not be sensitive.

### Dengue virus specific RT-PCR

Dengue virus RNA was extracted from serum using QIAmp viral RNA mini kit (Qiagen). RNA was reverse transcribed and the PCR was performed by using primer and conditions as previously described [18]. When determining the serotype of the infecting DV, positive controls for DEN-1, DEN-2, DEN-3 and DEN-4 were used in all experiments.

### Statistical analysis

Statistical analysis was performed using Graph pad PRISM version 4. As the data were not normally distributed, differences in means were compared using the Mann–Whitney *U* test (two tailed). Degree of association between clinical parameters and disease severity was expressed as the odds ratio (OR), which was obtained from standard contingency table analysis by Haldane’s modification of Woolf’s method. The Fisher’s exact test was used to determine the p value. Receiver-operator characteristic (ROC) curves showing the area under the curve (AUC) were generated to determine the discriminatory performance of platelet counts, lymphocyte counts and liver transaminase levels.

## Results

### Clinical parameters associated with severe dengue

The mean age the study participants was 27.18 (SD ± 11.74, range 15 to 68 years. 58 (31.8%) complained of abdominal pain and 31 (16.8%) had at least one bleeding manifestation. Table 1, describes the presence of WHO warning signs in patients with severe dengue and non severe dengue. Based on the 1997 WHO classification all our patients would have been classified as having dengue haemorrhagic fever (DHF) because all of them had thrombocytopenia (100,000 cells/mm^3^ or less) and a rising HCT which was defined as a HCT increase of ≥20% from the baseline of patient or population of the same age [19]. Of the 31 patients who developed bleeding manifestations in the absence of shock, 15 had petechie, 12 had ecchymosis, two had epistaxis, eight had per vaginal bleeding in the absence of their monthly period, four had haematemesis and three had melena. In the 2011 WHO guidelines, fever accompanied by a haemorrhagic manifestation along with thrombocytopenia and haemoconcentration is considered as diagnostic as DHF [3]. However, since this study was carried out in mid 2011, the tourniquet test was not carried out on any patients as it was not mentioned under warning signs in the 2009 WHO dengue guidelines [8].

**Table 1:** Clinical and laboratory parameters in patients with severe dengue and in those with dengue with warning signs who did not develop severe dengue

**WHO warning sign**	**Presence in patients with non severe dengue**	**Presence in patients with severe dengue**	**Odds ratio and CI**	**P value**
	**N = 144 (%)**	**N = 40 (%)**		
Abdominal pain	42 (29.6)	16 (40)	1.69 (0.7820 to 3.352)	0.24
Vomiting	57 (39.6)	20 (50)	1.526 (0.7547 to 3.087)	0.28
Clinical fluid accumulation	23 (15.9)	11 (27.5)	2.094 (0.9186 to 4.775)	0.10
Mucosal bleeding	18 (12.5)	9 (22.5)	2.03 (0.8332 to 4.957)	0.13
Lethargy/restlessness	1 (0.07)	2 (0.05)		
Increase in HCT	144 (100)	40 (100)		
Reduction in platelet counts <100,000cells/cm3	144 (100)	40 (100)		

### Presence of dengue warning signs and severity of dengue

All patients with severe dengue and non severe dengue had at least one dengue warning sign. Of the patients with severe dengue, 5 (12.5%) had five dengue warning signs, 4 (10%) had four dengue warning signs, 8 (20%) had 3 dengue warning signs, 8 (20%) had two dengue warning signs and 15 (37.5%) had at least one dengue warning sign. Of the patients with non severe dengue, 4 (2.78%) had five dengue warning signs, 15 (10.4%) had four dengue warning signs, 20 (13.9%) had three dengue warning signs, 46 (31.9%) had two warning signs and 67 (45.2%) had only one dengue warning sign. The presence of five or more dengue warning signs was significantly (p = 0.02) associated with the development of severe dengue (odds ratio 5.14, 95% CI = 1.312 to 20.16). The presence of two warning signs or less was significantly associated (p = 0.046) with a reduced risk of developing severe dengue (odds ratio 0.45, 95% CI 0.2163 to 0.9401). Of the patients with severe dengue 23 (57.5%) had only one to two warning signs. However, 113 (76.3%) of those with non severe dengue had only one to two warning signs. Therefore, it appears that patients with non severe dengue had fewer dengue warning signs when compared to those with severe dengue.

The presence of abdominal pain, vomiting, the presence of clinical fluid accumulation, mucosal bleeding were higher in patients with severe dengue. However, a statistically significant increase in patients with severe disease was not observed for any of the WHO warning signs. Although the odds ratio was >1 for all of the warning signs, they had a poor positive predictive value. For instance, the positive predictive value for abdominal pain was 40%, vomiting 50%, clinical fluid accumulation 27.5% and for mucosal bleeding 22.5%. However, the AST levels were significantly higher (p = 0.0001) in patients with abdominal pain (mean 243.5, SD ± 200.7), when compared to those who did not have abdominal pain (mean 148.5, SD ± 218.6). No significant difference (p = 0.56) was seen for ALT levels in patients with abdominal pain and those who did not have abdominal pain.

### Laboratory parameters associated with severe dengue

The laboratory parameters presented in the WHO warning signs are a rise in the HCT and a concurrent drop in the platelet counts. However, the guidelines highlight that full blood counts should be done at the first visit and that progressive leucopenia and rapid decrease in platelet counts usually precede plasma leakage [8]. Since all our patients who had severe dengue and non severe dengue had a rising HCT and falling platelet counts, we proceeded to analyze the usefulness of other simple parameters in patients with severe dengue and those who did not have severe dengue. The differences in laboratory parameters in those with severe dengue and the other patients are shown in Table 2. The platelet counts were significantly lower (p = 0.002) in patients with severe dengue, AST levels were significantly higher (p = 0.009). The ALT levels were not significantly higher (p = 0.09) in patients with severe disease. Although statistically not significant, the total leucocyte counts and the lymphocyte counts were lower in patients with severe disease when compared to those who did not develop severe disease.

**Table 2:** Laboratory parameters in patients with severe dengue and those who did not develop severe dengue

	**Severe dengue**	**Those who did not have severe dengue**	**P value**
	**N = 40**	**N = 144**	
	**Mean (±SD)**	**Mean (±SD)**	
Platelet counts	24653 (19508)	36343 (23653)	**0.002**
Total white cell counts	2854 (1437)	3550 (2260)	0.067
Total lymphocyte counts	1201 (853.0)	1674 (1554)	0.058
Total Neutrophil counts	1501 (1501)	1611 (955.7)	0.75
Aspartate transaminase levels	409.7 (912.5)	164.6 (158.9)	**0.009**
Alanine transaminase levels	194.4 (239.2)	126.7 (127.7)	0.096

Since low platelet counts, high AST levels and low lymphocyte counts appear to be associated with severe dengue, we went on to analyze if any of these tests could be used to predict the development of severe disease. We found that lymphocyte counts <1,500 cells/mm^3^ was significantly (p = 0.005) associated with severe clinical disease (odds ratio 3.367, 95% CI 1.396 to 8.123 (Table 3). The positive predictive value of a lymphocyte counts <1,500 cells/mm^3^ was 82.5%, with a specificity of 89.5% and a sensitivity of 28.2%. However, the lymphocyte counts had poor discriminatory value in predicting severe dengue as the AUC was 0.6 (95% CI 0.5071 to 0.6974). Although the total white cell count was lower in patients with severe dengue this was not statistically significant. There was no difference in the neutrophil counts in patients with severe dengue and non severe dengue (Table 2). Therefore, lymphopenia (lymphocyte counts <1,500 cells/mm^3^) rather than leucopenia, could be a more useful predictive factor of severe dengue.

**Table 3:** Association of laboratory parameters with severe dengue infection

**Clinical feature**	**Severe Dengue**	**Non severe dengue infection**	**Odds ratio and CI**	**P value**
	**N = 40**	**N = 144**		
Lymphocytes <1500	33	84	3.367 (1.396 to 8.123)	**0.005**
White cell count < 4000	33	105	2.389 (0.8688 to 6.567)	0.091
AST >45 IU	40	105	27.26 (1.632 to 455.2)	**<0.0001**
AST > 90 IU	25	76	1.491 (0.7265 to 3.061)	0.29
AST >225 IU	14	30	2.046 (0.9528 to 4.394)	0.09
Platelet counts <50,000/mm	34	101	2.413 (0.9437 to 6.168)	0.069
Platelet counts <20,000/mm	21	20	6.742 (3.089 to 14.71)	<0.0001

Severe organ impairment (AST or ALT >1000) is one of the criteria of severe dengue in the new WHO guidelines [8]. Therefore, we proceeded to determine the usefulness of liver enzymes in predicting those who are likely to develop severe dengue. All patients with severe dengue had high AST levels (>45 IU). High AST levels were also significantly associated (p < 0.0001) with severe disease (odds ratio 27.26, 95% CI 1.632 to 455.2) (Table 3). The wide CI for the odds ratio could be due to a small sample size (184 patients) and also possibly because none of the patients with severe dengue had AST levels <45 IU. The positive predictive value of a high AST (>45 IU) was 100%, with a specificity of 100% and a sensitivity of 27.6%. However, the AST levels did not have a good discriminatory value is predicting those with severe dengue as the AUC was 0.65 (95% CI 0.5486 to 0.7436).

Platelet counts <20,000 cells/mm^3^, was again significantly associated (p < 0.001) with severe disease (odds ratio 1.632 to 455.2, 95% CI 3.089 to 14.71) (Table 3). Again, platelet counts alone did not appear to have a good discriminatory value in predicting those with severe dengue (AUC 0.66). However, the combination of the presence of 4 or more dengue warning signs, platelet counts <50,000 cells/mm^3^ and the presence of lymphocyte counts <1,500 cells/mm^3^, was significantly (p = 0.002) associated with the development of severe dengue (odds ratio 5.18, 95% CI 1.917 to 14.00). The reason we chose platelet counts <50,000 cells/mm^3^ for the analysis of this purpose was that by the time the platelet counts dropped to <20,000 cells/mm^3^ in those with severe dengue, they were already critically ill.

### Virus serotypes isolated from our patients

It was possible to obtain a blood sample during the first 5 days in 84/184 of the individuals included in the study. RNA was extracted from the serum of these patients and RT-PCR for dengue was carried out. The PCR was positive in 26/84 of the patients and we found that the infecting serotype was DEN-1 in all 26 patients. The positive controls for DEN-2, DEN-3 and DEN-4 worked in all experiments.

## Discussion

The WHO changed their dengue disease classification and management guidelines in year 2009 in order to develop criteria which could be used to clearly differentiate severe dengue from non severe dengue. In fact the new classification criteria were found to be far more sensitive and specific in detection of patients with severe dengue, when compared to the traditional criteria [15,20]. After carrying out extensive multicentre studies, simple clinical signs and symptoms, which would enable medical personal to clearly distinguish between severe and non severe forms of dengue were developed [14]. The objective of the use of warning signs was to enable health care professionals to decide who needs hospital admission and more intensive monitoring during epidemics of dengue infection. Although these warning signs have immensely helped to decide who needs hospital admission, due to the large numbers of individuals affected during an epidemic of dengue, massive numbers of patients are admitted to our hospital every day. However, in resource poor setting such as in Sri Lanka, intense monitoring of all patients admitted to hospital with dengue infection is impossible due to the large numbers. Therefore, we attempted to define the usefulness of the new WHO warning signs in predicting those who are likely to develop severe dengue in adult patients admitted to hospital with acute dengue infection.

We found that the presence of all the WHO warning signs such as abdominal pain, vomiting, clinical fluid accumulation were higher in patients with severe dengue. Both abdominal pain and persistent vomiting are considered as warning signs in the revised WHO disease classification. Many patients are known to present with abdominal pain [21,22] and it has been shown to be associated with severe dengue [23,24]. Some patients have been shown to present with symptoms predominantly related to gastrointestinal symptoms such as vomiting, diarrhoea and abdominal pain, which were associated with a poor outcome [22,24]. Importantly, we found that the presence of abdominal pain was significantly associated with raised AST levels. Therefore, the presence of abdominal pain in a patient with dengue appears to be associated with liver involvement. The presence of five or more dengue warning signs was significantly (p = 0.02) associated with the development of severe dengue (odds ratio 5.14, 95% CI = 1.312 to 20.16). Therefore, although all warning signs are important, we found that the presence of five or more warning signs should particularly alert the physician of a higher possibility of severe dengue in such patients.

The 2009 WHO guidelines include a high or progressively rising haematocrit concurrent with a rapidly decreasing platelet count as warning signs. Although the guidelines do not highlight leucopenia are as warning sign, it is mentioned that progressive leucopenia and rapid decrease in platelet counts usually precede plasma leakage, In addition to leucopenia, we found that lymphocyte counts <1,500 cells/mm^3^, platelet counts <20,000/mm^3^ and raised AST levels were associated with severe dengue and have better positive predictive values in predicting severe dengue when compared to clinical parameters alone. Since no difference was observed in the neutrophil counts in those with severe dengue and non severe dengue, it appears that lymphopenia is contributing to severe dengue. However, none of these laboratory parameters when used alone had a good discriminatory value in predicting those with severe dengue.

The warning signs in 2009 WHO guidelines do provide advantage in resource poor countries where it is sometimes not feasible to carry out laboratory investigations and is extremely useful to decide who needs hospital admission [25]. However, there has been some criticism regarding the 2009 WHO dengue guidelines, as some experts believe that it has actually increased the number of hospital admissions and thus the work load. There have been suggestions that laboratory parameters such as plasma leakage detected by haemoconcentration should be included as a major criterion [26]. Many have suggested that more emphasis should be placed on plasma leakage as the main underlying problem in severe dengue [27]. During epidemics of dengue infection large numbers of patients are admitted to hospital and it is almost impossible to closely monitor all patients due to scarcity of resources. Therefore, we feel that simple laboratory tests such as full blood counts and AST levels could help in determining those who are likely to develop severe dengue so that clinicians can determine the patients who are most likely to need closer monitoring and more intensive treatment after admission to hospital. Studies done in children with acute dengue also have shown that leucopenia (<4000 cells/mm^3^) was associated with the development of shock [28]. Both lymphocyte counts and white cell counts have been shown to be significantly lower in patients with acute dengue infection who were hospitalized, when compared to those who were cared for as out-patients [29]. We too found that lymphocyte counts <1,500 cells/mm^3^ was significantly (p = 0.005) associated with severe clinical diseases (odds ratio 3.367, 95% CI 1.396 to 8.123). The positive predictive value of a lymphocyte counts <1,500 cells/mm^3^ was 82.5% in predicting severe dengue. Although reduction of platelet counts are mentioned as a warning sign, we found that platelet counts were more strongly associated with severe dengue when they reduced to <20,000cells/mm^3^. However, none of the laboratory parameters on their own had a good discriminatory value in predicting severe dengue. Therefore, we assessed if a combination of clinical and laboratory parameters could be used to predict severe dengue. We found that the combination of the presence of 4 or more dengue warning signs, platelet counts <50,000 cells/mm^3^ and the presence of lymphocyte counts <1,500 cells/mm^3^, was significantly (p = 0.002) associated with the development of severe dengue (odds ratio 5.18, 95% CI 1.917 to 14.00).

Raised AST levels were detected in all patients with severe dengue and AST levels were significantly higher in patients with severe dengue. Hepatic involvement and dysfunction is a well recognized complication of dengue [30,31]. Hepatic dysfunction also leads to deranged coagulation profiles, which could contribute to bleeding in dengue infections [31,32] and is associated with a high mortality [30,33,34]. In the 2009 WHO guidelines, severe dengue is also classified by the presence of AST values >1000 IU. Although very correctly AST levels are not included as a warning sign in the 2009 guidelines as it would be a waste of resources to carry out AST levels in all patients with suspected dengue, we feel that once patients are admitted to hospital, it would be a useful test to determine those needing more intense monitoring and indeed is recommended in the guidelines to detect those with AST > 1000 IU. Potts et al. have also highlighted the usefulness of inclusion of AST values >50 IU along with leucocyte and platelet counts in correctly classifying individuals who are likely to develop severe dengue [35]. We too feel that it would be useful to include both the AST levels (below 1000 IU) and lymphocyte counts <1,500 cells/mm^3^ in the warning signs as these are very simple laboratory tests to carry out and would help in patient triage in resource-poor settings.

Dengue infection is caused by four dengue virus serotypes (DEN1-4) which are closely related. Although all four serotypes have been circulating in Sri Lanka, DEN-2 and DEN-3 were the predominant serotypes isolated from patients with acute infection until year 2009. These 2 serotypes were responsible for 86% of acute dengue infections whereas DEN-1 serotype only accounted for 7% of infections [36]. However, a new strain of the DEN-1 was identified in Sri Lanka for the first time in year 2009, which coincided with the dengue epidemic in year 2009. DEN-1 was the only virus serotype identified among patients in our cohort, which probably indicates that DEN-1 was the predominant circulating serotype during this period. The introduction of a new DEN-1 virus strain to Sri Lanka probably resulted in a shift in the predominant circulating virus serotype and thus caused a massive dengue epidemic in non immune individuals.

## Conclusion

In summary, we have attempted to determine clinical and laboratory parameters that would help us to predict severe dengue infections and the usefulness of some of the warning signs included in the revised WHO clinical disease severity classification. We found that the presence of all the WHO warning signs such as abdominal pain, vomiting, clinical fluid accumulation were higher in adult patients with severe dengue and are useful signs and that the presence of 5 or more dengue warning signs was significantly associated with the development of severe dengue. We also found that lymphocyte counts <1,500 cells/mm^3^, platelet counts <20,000/mm^3^ and raised AST levels were associated with severe dengue. However, as this study was restricted to hospitalized adult patients with dengue infection, the predictive values and usefulness of these clinical and laboratory parameters may differ in children and also in adult patients with less severe forms of dengue. Furthermore, although we present data on a cohort of 184 individuals, it will be important to increase patient numbers in future studies in order to stratify groups and minimize confounding factors.

The predominant circulating DV serotype during the year 2011 appears to be DEN-1, which was in contrast to the pattern observed before year 2009. Therefore, the shift in the predominant circulating DV serotype would have resulted in the massive dengue epidemics that occurred in Sri Lanka during years 2010 and 2011.

## Ethical approval

This study was approved by the Ethical Review Committee of the University of Sri Jayawardenapura.

## Competing interests

The authors declare that they have no competing interests.

## Authors’ contributions

SDJ, GNM, GSO: the conception and design of the study, drafting the manuscript.TC: Design of study and revising the manuscript critically for intellectual content. VA, LG, SF, TW: acquisition of data, analysis and of data. All authors have seen and given the approval of the final draft.
